# Laryngeal Paraganglioma—A Case Report

**DOI:** 10.3390/medicina60020198

**Published:** 2024-01-24

**Authors:** Dragos Octavian Palade, Florentina Severin, Daniela Vrinceanu, Razvan Hainarosie, Alma Maniu, Huzafa Ahmed, Felicia Manole, Florin Mocanu, Catalina Voiosu

**Affiliations:** 1 Faculty of Medicine, “Grigore T. Popa” University of Medicine and Pharmacy, 700115 Iasi, Romania; octavian.palade@umfiasi.ro (D.O.P.); florentina-s-severin@umfiasi.ro (F.S.); huzafa.ahmed@email.umfiasi.ro (H.A.); 2 ENT Department, Faculty of Medicine, “Carol Davilla” University of Medicine and Pharmacy, 020021 Bucharest, Romania; daniela.vrinceanu@umfcd.ro (D.V.); razvan.hainarosie@umfcd.ro (R.H.); catalina.pietrosanu@umfcd.ro (C.V.); 3 Department of Otolaryngology, “Iuliu Hatieganu” University of Medicine and Pharmacy, 400347 Cluj, Romania; aurelia.maniu@umfcluj.ro; 4 Faculty of Medicine and Pharmacy, University of Oradea, 410073 Oradea, Romania

**Keywords:** larynx, paraganglioma, tumor, surgery

## Abstract

*Background and Objectives*: Paragangliomas of the head and neck are rare neuroendocrine tumors originating from the paraganglia, which might be sympathetic or parasympathetic. Laryngeal paragangliomas are the rarest subtype of these tumors, with only 1.41% of all paragangliomas, arising from the supraglottic or subglottic paraganglia of the larynx. The vast majority of them are benign, but there are some cases in which they turn out to be malignant, and the only way to know with certainty the difference between them is when we identify distant metastases. The aim of this article is to share our experience with a rare case of laryngeal paraganglioma and review the clinical characteristics, methods of diagnostic, necessary investigation prior to the operation, and surgical management of this type of tumor. *Materials and Methods*: We present the case of a 68-year-old female patient, a non-smoker, who accused dysphagia, dysphonia, foreign body sensation, chronic cough, and hoarseness for six months. We performed a tracheostomy prior to biopsy to secure the airways in case of bleeding and then took a few biopsy samples. The histopathological exam revealed the presence of a laryngeal paraganglioma. An enhanced CT scan was performed in order to describe the localization, size, and invasion of the tumor. We also measured the vanillylmandelic acid from the urine to determine if the tumor produced catecholamines alongside a full cardiology and endocrinology examinations. In order to prevent massive bleeding during the operation, chemoembolization was attempted before surgery, but it was unsuccessful due to an anatomical variation of the left superior thyroid artery. She underwent surgery, first through transoral endoscopic microsurgery; however, we decided to undertake an external approach because of poor bleeding control, even though we had ligated both the superior thyroid artery and the external carotid artery, with a thyrotomy and laryngofissure achieving the complete resection of the tumor. *Results*: The patient was discharged 10 postoperative days later, with the recommendation of introducing food step-by-step from liquids to solids. She was decannulated after 30 days, with no complications regarding breathing, phonation, or deglutition. Twelve months after the surgery, we did not identify any local relapses of distant metastases. *Conclusions*: Laryngeal paragangliomas are rare neuroendocrine tumors that arise from the laryngeal paraganglia. Surgery is the best treatment option available, and it can be done by either an external approach or by transoral endoscopy. Enhanced CT or MRI, as well as full cardiological and endocrinological evaluation are mandatory prior to the operation. Measuring the catecholamines levels show the if the tumor is secretory. Controlling the bleeding poses the biggest challenge in performing the resection of the tumor, especially when a transoral endoscopic approach is chosen. Further standardized follow-up guidelines are required in the future.

## 1. Introduction

Head and neck paragangliomas are tumors of a neuroendocrine origin that develop from the paraganglia in the head and neck. They are diverse, rare, and the vast majority of them are benign. They originate from the chromaffin cells from the paragangliomic tissue of the autonomic parasympathetic nervous system [1].

Paraganglia cells have their embryological origin in the neural crest cells that move within the autonomous nervous system [2]. Head and neck paragangliomas represent between 65% and 70% of all cases of paragangliomas and are responsible for almost 0.65% of all neoplasms of the head and neck [3]. Their total incidence in the general population is estimated to be between 0.3 and 1 per 100,000 [3]. A female predominance has been observed with a ratio of 3:1 [2]. About 95% of the tumors are non-secretory, mostly being solitary, unilateral, and appearing at ages between 40 and 70 [4]. They are less likely to arise from the cervical paraganglia, with most of them originating from the body the carotid artery or vagal nerve glomus [4]. Adding to the afore-mentioned points, they can arise from glossopharyngeal paraganglia and jugulo-tympanic PGLs, but they can also be found in more particular locations such as the larynx, nasal cavity, trachea, thyroid, or the orbit [5].

The majority of paragangliomas are benign, but about 10% of them are malignant [2]. The difficulty in differentiating those that are malignant from those that are benign is due to the fact that there are no histopathological or immunohistochemical criteria and there only is the identification of distant metastases available to us [2]. Sinonasal paragangliomas have the highest rate of malignancy of about 24%, after that vagal PGLs have the next highest rate (10%), that of jugulo-tympanic paragangliomas is 5.1%, that of carotid artery PGLs is 1.41%, and laryngeal paragangliomas are the least likely to become malignant, with only 1.36% of them becoming so [6].

When talking about genetic factors that influence the development of this type of tumor, it has been revealed that 35% of head and neck paragangliomas have a familial component [1]. Head and neck paragangliomas appear to be linked to the mutations of the succinate dehidrogenase complex (SHD) [7]. Heredity of paragangliomas has been proved to be involved in a higher risk of metastases than that which has been observed in those with no familial history of them, and the most encountered mutation in patients with malignant paragangliomas is that of succinate dehidrogenase subunit B [8]. Unfortunately, the survival rate of patients who present distant metastases is estimated to be around 50% upon 5 years [8].

Clinical characteristics of the tumors depend on the location in which they occur, with most of them being asymptomatic. Laryngeal paragangliomas, the least encountered of all of them, represent only 1.41% of all head and neck paragangliomas [6]. They originate from the superior and inferior laryngeal paraganglia, with 90% of them being supraglottic [9]. Patients with laryngeal paraganglioma can present dysphagia, dyspnea, hoarseness, and stridor [10]. Nonetheless, these symptoms are not only specific to the paragangliomas that develop in the larynx. Paragangliomas that form from the vagal nerve in the parapharyngeal space, especially tumors that develop behind styloid process, cause hoarseness due to vocal fold paralysis [11]. Laryngeal paragangliomas pose a difficult challenge when differentiating them from other laryngeal tumors. The immediate threat that they pose is that to the airway; operating on them, either through a conservative approach or through an external one, raises difficulties in avoiding injures to the larynx and pharynx and having proper bleeding control in place.

Histological examination represents the gold standard for the diagnosis and differentiation of a paraganglioma from other cervical neoplasms. It is represented by a particular aspect called the “Zellballen” pattern consisting of well-developed tumoral cells that gather in a nest-like form associating with fibrovascular tissue and peripheral sustentacular cells in the stroma [12]. When it comes to immunohistochemical markers, it is important to measure the marker of proliferative activity, Ki67, that is associated with other features such as a tumor bigger than 5 cm, the presence of necrosis, SHDB mutation, and an immature secretory phenotype, which might suggest the malignant character observed [13]. The most common places for metastases are the lungs, bones, liver, and lymph nodes [7].

Imaging also plays a crucial role in identifying and describing paragangliomas of the head and neck. Current CT and MRI provide the best options in the evaluation of tumors, with each of them offering specific advantages depending on the case. While MRI offers a more accurate description regarding the tumors involving the soft tissues, CT represents a better option when it comes to the evaluation of the extension to the temporal bone in cases of jugular glomus or glomus tympanicum paragangliomas [14].

Treatment is adapted to the behavior and localization of the tumor. For the benign head and neck paragangliomas that show no invasive behavior, surgery with large margins of resection improved the survival rate, with no adjuvant treatment needed, and a decreased chance of recurrence was demonstrated as well [15]. In patients with head and neck paragangliomas that prove to either have an invasive behavior or recurrence after a previous surgery, the association of surgery and radiotherapy proved to give a higher rate of survival [15]. When it comes to laryngeal paragangliomas, some aspects, such as the risk of bleeding, should be taken into consideration due to the fact that they are characterized by hypervascularization and some of them might secrete catecholamine. Appropriate measures should be taken, such as proper imaging of the vascularization of the tumor through CT or MR angiography and, if possible, embolization of the tumor’s blood supply [16]. The process of preparing the patient for surgery must include an imaging examination to establish the size and the invasion of the tumor, as well as the structures with which the tumor has close contact. Any comorbidities that might prevent the operation being carried out well must be addressed; therefore, a multidisciplinary examination is mandatory, with emphasis placed on cardiological and endocrinological pathologies. When talking about laryngeal paragangliomas, we should take into consideration the possibility of an injury that might permanently affect the phonation of the patient. The patient should be told about this unfortunate outcome and assess the possible vocal prosthesis and voice rehabilitation after the tumor is resected [17].

## 2. Case Presentation

A 68-year-old female was presented to our clinic with dysphagia for hoarseness, aspiration of saliva, chronic cough, and dysphonia for six months. She did not show any associated comorbidities or undergo regular treatment, with normal blood pressure values. The patient had no history of smoking. No familial history of oncological pathologies was uncovered.

**Figure 1:**
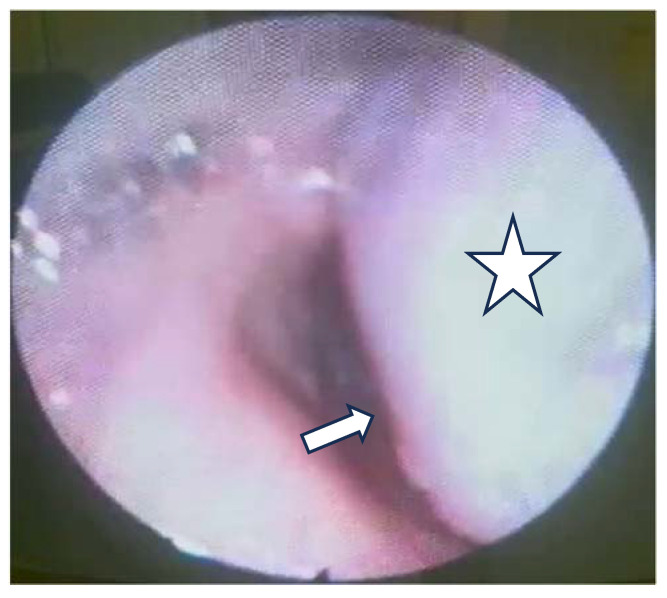
The tumor bulges into the laryngeal inlet (the laryngeal inlet is marked by the arrow and the tumor by a star).

The flexible video laryngoscopy examination revealed a supraglottic mass covering the left aryepiglottic fold and the left arytenoid cartilage, extending to the ipsilateral pyriform sinus. The tumor presented a pronounced vascular draw, having a blueish color. The tumor deviated from the left hemilarynx medially, thus decreasing the movement of the left true vocal fold. The tumor tilted over the laryngeal inlet’s opening, causing breathing difficulties. The right vocal fold has a normal aspect, with normal movement, while the tumor did not obstruct the glottic space (Figure 1).

**Figure 2:**
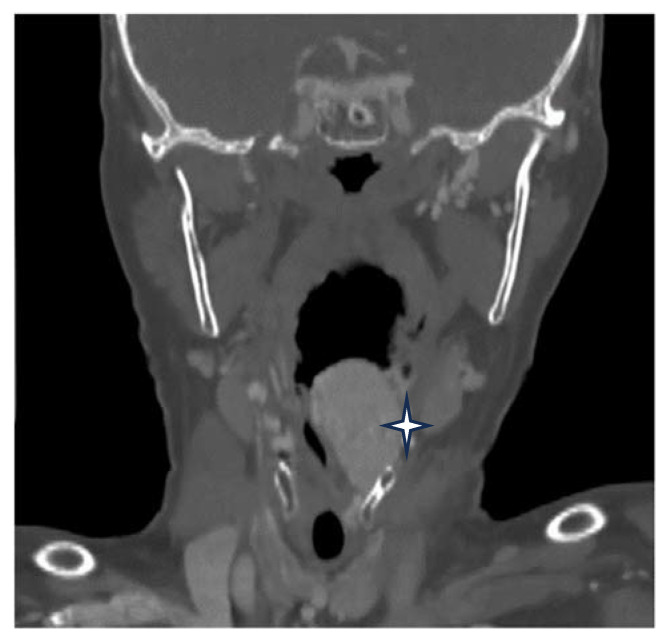
Enhanced CT scan of the tumor (marked by star).

A postcontrast computer-tomography (CT) scan of the neck revealed an expanding tumor, which was intensely enhancing and heterogenous, with dimensions of 28/24 mm in diameter. It had mainly developed inside the hypopharynx and larynx and centered itself at the left pyriform sinus that deviates the epiglottis, embedding itself in the left aryepiglottic fold, the arytenoid cartilage, and the false vocal fold, and bulging into the laryngeal inlet, thereby coming into close contact with the right arytenoid cartilage. At the upper pole, it extended from the greater horn of the hyoid bone along the entire length of the thyroid cartilage, down to a line that passed across the level as that of the arytenoid cartilage. The CT scan bilaterally objectified the presence of some enlarged cervical lymph nodes in group III (Figure 2).

Due to the aspects of the tumor, multiple diagnoses were considered as follows: a vascular tumor, a schwannoma, a laryngeal carcinoid, or a paraganglioma. In our case, considering the size, given the fact that the tumor bulged into the laryngeal inlet alongside the high risk of bleeding, the decision was made to undertake the tracheostomy before taking a biopsy.

**Figure 3:**
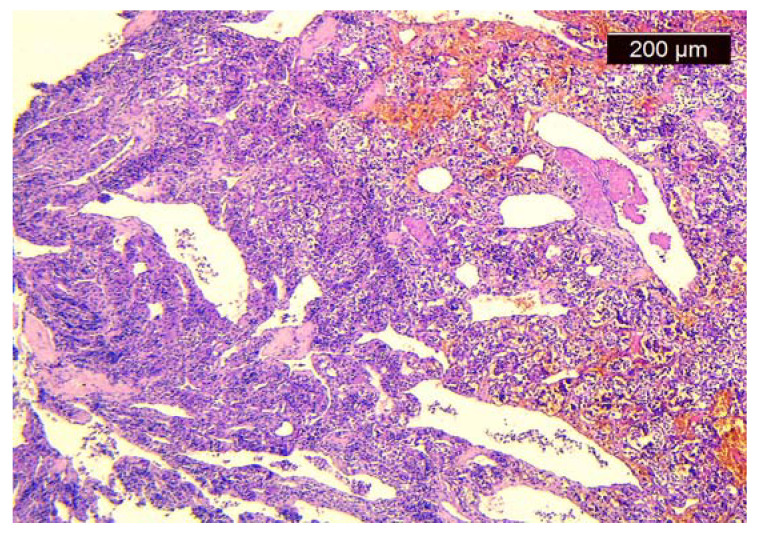
Histological aspect of the tumor.

We took multiple pieces from the tumor with the help of a flexible endoscope with a working channel, preserved them in formalin recipient, and handed them to the anatomopatology department. The biopsy revealed the absence of dysplasia, the presence of the “Zellballen” pattern, cubic cells gathered in nest-like groups, rich vascularization, and a Ki67 of 50%, all suggesting a laryngeal paraganglioma (Figure 3).

Considering the nature of the tumor, the patient underwent a complete cardiological and endocrinological examination. None of the aforementioned examinations revealed any comorbidities. It is worth mentioning that the patient had her vanillylmandelic acid measured from urine, the values of which were, in case there was a tumor present that produces catecholamines. Vanillylmandelic acid is a major product from the metabolization of norepinephrine and epinephrine and is excreted through urine. Its high level might have suggested a secretory tumor, which was not the case here.

The size, localization, and type of tumor made us decide to undertake the chemoembolization of the left superior thyroid artery before the surgery to reduce the risk of bleeding. Unfortunately, the attempt failed due to an anatomical particularity of the artery; therefore, ligation of the left superior thyroid artery was taken into consideration, which involved exposing the external carotid artery and placing a thread within this level in case of there was a massive hemorrhage.

**Figure 4:**
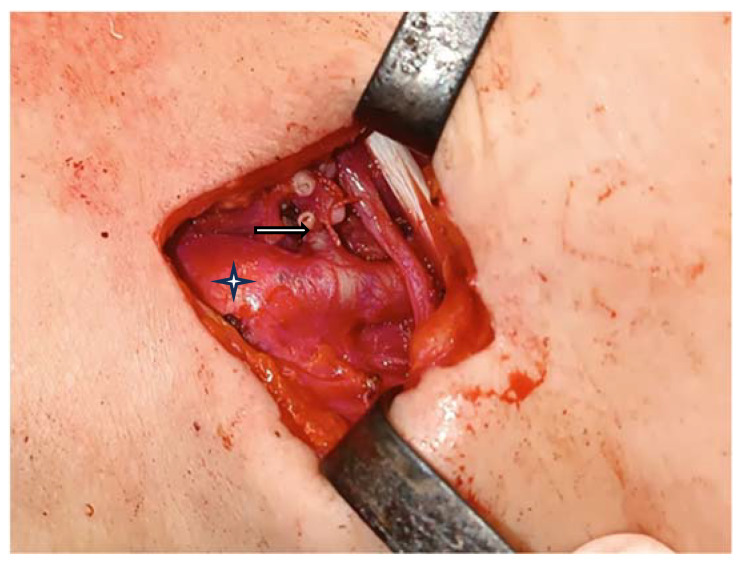
Sectioning and ligation of the left superior thyroid artery (white arrow) and the external carotid artery (pointed to by the star).

The left superior thyroid artery was exposed, divided, and ligated (Figure 4). The decision was to first try the excision through a transoral endoscopic approach with radiofrequency instruments. We performed a suspension laryngoscopy. With a radiofrequency fine needle, we attempted the resection of the tumor starting from its origin at the pyriform sinus, and we tried to protect the larynx as much as we could. Unfortunately, the bleeding was impossible to control, even after the ligation of the external carotid artery, so an external approach was chosen.

**Figure 5:**
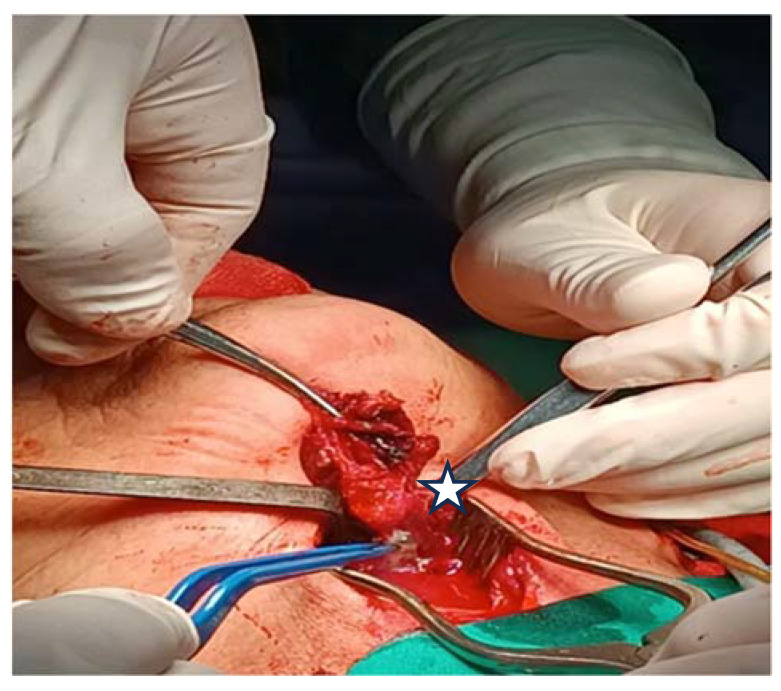
Resection of the tumor (marked by the star).

**Figure 6:**
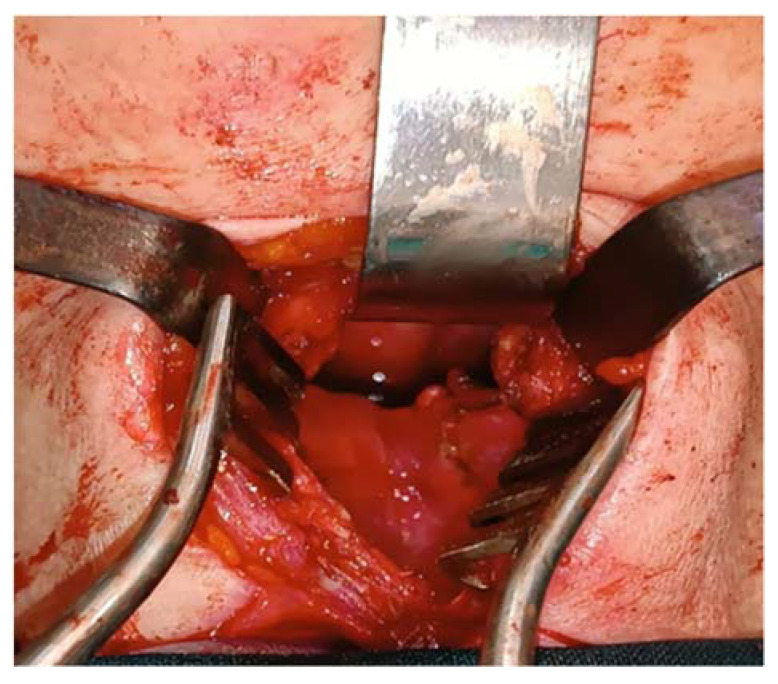
The view of the larynx after resection of the tumor.

A vertical incision on the anterior midline of the neck was made, with dissection of the deep layers, retraction of the strap muscles, and exposure of the thyroid cartilage all undertaken. A vertical incision along the midline of the cartilage and a laryngofissure were performed, and the two flaps were retracted, thus exposing the tumor. Using bipolar cautery and blunt instruments, the tumor was resected, and the local bleeding was stopped while we simultaneously took care not to cause any unnecessary injuries to the larynx or the pharynx (Figure 5 and Figure 6). We removed the thread from the external carotid artery before closing the thyroid to check if any bleeding might occur and to prevent any unnecessary complications that might appear.

**Figure 7:**
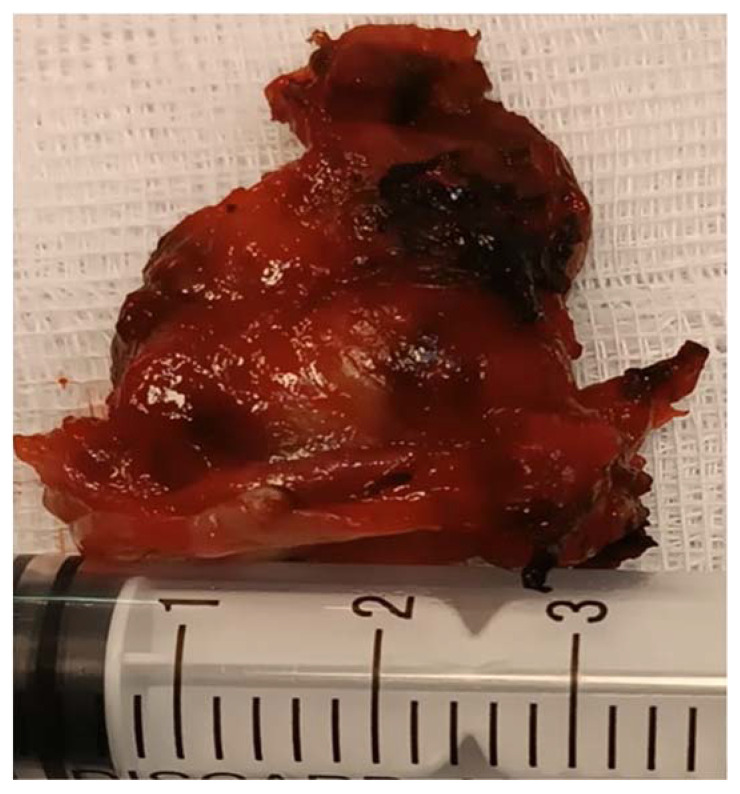
The resected tumor.

A tumor of 30/30 mm was resected and was sent to the anatomic pathology department (Figure 7). The histological result confirmed the diagnosis of a laryngeal paraganglioma.

The patient lost approximately 400 mL of blood; therefore, it was decided that a transfusion of a 500 mL blood bag was needed. The blood analysis from the next day confirmed hematocrit, hemoglobin, and a number of red cells within normal values. During the hospitalization, a nasogastric tube was fitted to feed the patient, to reduce the stress that the deglutition effort might cause to the larynx and pharynx, and to accelerate the healing.

After 7 days, the flexible video laryngoscopy showed that the surgical defect had healed well enough to remove the nasogastric tube and allow the patient to eat liquid food. Upon the 10th postoperative day, we decided to discharge the patient with the strong recommendation of only introducing solid food after another 15 days for a better healing process and to avoid any perforations of the left pyriform sinus that might occur. Due to the moderate laryngeal edema that was still present, we decided to maintain the tracheal tube for another 30 days.

After 1 month, the flexible video laryngoscopy revealed that the larynx had healed entirely. The patient did not present any hoarseness, dysphagia, or stenosis of the airways; therefore, the tracheostomy was removed. The true vocal folds had a regular aspect, and their movement was preserved. Moreover, the patient did not show any phonation problems.

**Figure 8:**
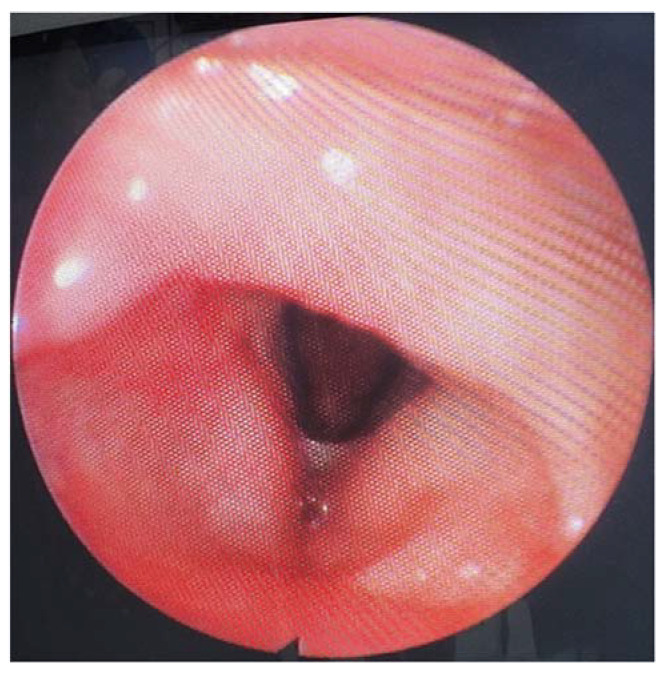
Postoperative view of the larynx after 3 months.

No radiotherapy was necessary because no relapse was observed at 30 days or at 3 months postoperatively (Figure 8).

We decided that the patient should undergo a flexible video laryngoscopy and a complete enhanced CT scan of the neck, thorax, and abdomen for any metastases every 6 months for the first 2 years. No relapses or distant metastases were uncovered after 12 months of monitorizations. 

The patient did not display any breathing, phonatory, or deglutition problems. The flexible video laryngoscopy undertaken at 12 months showed us that the larynx was covered by normal mucosa, with preserved mobility of the vocal folds and no sign of relapse or any scarring postoperatively (Figure 8).

## 3. Discussion

Head and neck paragangliomas are a rare group of tumors, representing a small percentage of all neoplasms that occur in this part of the body; therefore, they constantly pose a challenge to every surgeon who has to deal with them, especially those who are in close contact with large vessels and cranial nerves, and for their resection has a high risk of devastating complications [18].

The lack of specific histological and immunohistochemical markers for differentiating between benign and malignant paragangliomas represents one of the biggest challenges in establishing the proper approach. Some factors, such as a high Ki67 of > 3%, the presence of central necrosis, and hypervascularity of the tumor, do not represent specific markers for malignancy. Therefore, according to the latest guidelines, they are all considered malignant [3].

Laryngeal paragangliomas are the least encountered of all head and neck paragangliomas, representing only 1.41% of all cases [6]. Patients diagnosed with laryngeal paraganglioma present dyspnea, dysphagia, hoarseness, chronic cough, and stridor. In most cases, they do not reveal symptoms specific to high levels of catecholamines, with most of the tumors being non-functional [10]. Our patient presented these aforementioned symptoms for almost 6 months. The first of these symptoms was the chronic cough, which was followed by hoarseness, dyspnea, and stridor. Dysphagia came last. These symptoms are common for any tumor that occurs at the laryngeal or pharyngeal level [19,20].

The first step in establishing a diagnosis is that of carefully examining the tumor through flexible video laryngoscopy. It helps us to describe the tumoral mass, localization, origin, size, which structures of the larynx it has invaded, and the necessity of a tracheostomy before the biopsy due to an expected significant bleeding or because it obstructs the upper airway. It also offers vital details for the surgical strategy, such as deciding whether the tumor can be resected through transoral endoscopic surgery or whether it requires an external approach by performing a laryngotomy with a thyrotomy. In our case, considering its size, the fact that the tumor bulged to the laryngeal inlet, and the high risk of bleeding, the decision to undertake the tracheostomy was made before taking a biopsy.

Histological and immunohistochemical exams represent the gold standard for diagnosing laryngeal paraganglioma by differentiating it from other neoplasms, such as carcinoid tumors or schwannomas. Although it lacks the possibility of establishing whether it is a benign or malignant tumor, it offers some clues in this direction by identifying the “Zellballen” pattern and measuring Ki67 [12]. We took several biopsy samples from the tumor using a flexible video laryngoscope with a working channel because we considered it to be more accurate due to video monitorization and to be more comfortable in case we needed to take more samples than that of a fine needle aspiration biopsy.

Before surgery, a multidisciplinary examination of the patient should be undertaken. In our case, the patient was examined for cardiological pathologies and any endocrinological abnormalities. When the tumor involves the vagal nerve or the great cervical vessel, the patient might sometimes necessitate an assessment undertaken by a neurologist or a vascular surgeon in case there are possible complications that might occur after surgery. Measuring metanephrine taken from blood and urine is important for a catecholamine-secreting tumor [21]. In our case, the patient had vanillylmandelic acid measured from the urine to rule out the possibility of a secretory tumor.

Radiological assessment is crucial in utilizing the proper therapeutic conduit. Imaging, such as enhanced CT, MRI, ultrasound, and angiography, are the best modalities used to describe the tumor, localization, size, and involvement of essential vessels [12]. Three-dimensional modelling represents a unique alternative in describing anatomical challenges and critical neurovascular structures, thereby providing a more comprehensive evaluation of the tumoral invasions or structures that are in close contact with the tumor [18]. When distinguishing between paragangliomas and other tumors, Ga-68 DOTATATE PET/CT proved reliable. Paraganglioma has an overexpression of SSTRs, thus providing them with better enhancement than that of tumors like schwannoma, meningioma, and esthesioneuroblastoma, which have a lower uptake [22]. Considering the fact that the tumor was located in the larynx, we decided that an enhanced CT scan of the neck and thorax would offer enough details regarding the size, localization, and extension of the tumor. In our case, the MRI could not provide any supplementary significant details but would have been more costly.

Laryngeal paragangliomas are described as neuroendocrine, highly vascularized tumors. Therefore, significant bleeding during surgery is expected. To prevent this complication, multiple methods were taken into consideration. Such tumors obtain their blood supply from the superior thyroid artery. Consequently, it is the target of the strategies used to prevent severe bleeding. The most obvious method is the ligation of the superior thyroid artery on the same side of the tumor or on both sides of it, but the newest method is that of embolization through super-selective catheterization of the branches alongside chemical transarterial embolization [23]. Another option is the direct devascularization of the tumor by injecting cyanoacrylate glue directly into the paraganglioma [24]. Unfortunately, injection of the glue into the tumor might result in the agent’s migration into intracranial circulation with fatal consequences [23]. In our case, the preoperative embolization of the superior thyroid artery was tried, but it was not possible due to an anatomical variation of its tract. First, we ligated the mentioned-above artery, but the bleeding was significant. Therefore, we went further by ligating the external carotid artery during the procedure, releasing it at the end with a reasonable level of control of the blood loss and no further complications.

In treating laryngeal paragangliomas, multiple methods have been described, with surgery being the gold standard, which is accompanied or not accompanied by radiotherapy [3]. We can divide the surgical techniques conservatively through the transoral endoscopic approach or invasively through a laryngotomy with a thyrotomy. The algorithm used to choose between any of these approaches depends on the size of the tumor, invasion into the surrounding structures, vascularization and the control of the bleeding, available equipment, surgeon experience, and possible complications [25].

First, a transoral endoscopic approach was tried. We used radiofrequency cautery instruments to resect the tumor, avoiding any unnecessary injuries to the larynx or the pharynx. Still, the bleeding control was poor despite a preceding ligation of both the superior thyroid artery and the left external carotid artery. Therefore, we proceeded with an external approach. We made the incision on the anterior cervical midline and exposed the thyroid cartilage, which was divided with the flaps being elevated. We exposed the tumor and, using a combination of cautery and blunt instruments, we managed to resect the tumor, control the bleeding, avoid any injuries with no postoperative complications, and preserve the laryngeal functions. As a result, the patient was decannulated after one month. Despite this, cases of laryngeal paragangliomas treated through the transoral endoscopic approach and laser resection have been reported [26]. We decided to attempt a transoral endoscopic approach in order to avoid injuries to the larynx and pharynx that might come with external surgery, such as the perforation of the pyriform sinus, fracture of the thyroid cartilage, injuries to the vocal folds or the arytenoid cartilages, and to avoid adding another scar for a faster healing of the patient and, of course, for esthetic reasons. The open approach, although more brutal, provides a better view and larger access to the tumor, easier ways to control the bleeding, but also involves the use of larger, more traumatic instruments. Moreover, the risk of infections is higher because we create new areas of contact with the outer environment through the open approach.

Radiotherapy is considered to be a viable option for patients with unresectable tumors due to their size, location, invasion into the surrounding structures, or because the health conditions of the patients would not allow them to undergo surgery [27]. In this case, the patient did not need radiotherapy because complete excision of the tumor was achieved, and evaluation after one month showed no relapse.

We first decided to reevaluate the patient after 30 days in order to remove the tracheostomy. The flexible laryngoscopy showed us that the larynx and pharynx had healed, with normal movements of the vocal fold and the patient being able to speak and eat normally. We decannulated the patient and established a follow-up plan for the first 2 years. During this time period, the patient would undergo an enhanced CT scan for the neck, thorax, and abdomen to uncover any possible metastases as well as a flexible video laryngoscopy for local relapses. We chose this plan using the follow-up plan for the oncological patients due to the lack of specific guidelines regarding paragangliomas.

Our case of laryngeal paraganglioma offers some possible solutions in managing blood control in case of a failed chemoembolization. We showed that ligating the superior thyroid artery and the external carotid artery might be necessary in order to avoid a massive hemorrhage. In this case, converting the operation from a transoral endoscopic surgery to an external approach that involves a thyrotomy and laryngofissure, was necessary to obtain a better level of bleeding control after the resection of the tumor. This shows us that surgery on paragangliomas presents significant challenges and that a team of surgeons must be ready to change the approach even during the operation. The disadvantage that we faced was that of the impossibility of a successful chemoembolization due to an anatomical variation that pushed us to use more invasive surgical solutions.

Considering the data that we gathered from the experience of other case reports of laryngeal paragangliomas and from our own experience, we predict that our patient has a very low chance of developing metastases or local relapses, but further follow-up guidelines should be developed. Due to the difficulty of differentiating benign paragangliomas from malignant ones, we suggest that they should be treated and observed as malignant ones until further specific protocols are developed.

## 4. Conclusions

Laryngeal paragangliomas represent the rarest of this type of tumor with an origin in the laryngeal paraganglia. Most of them are benign, but a small percentage of them become malignant.

The lack of specific histological and immunohistochemical specific makes their specific differentiation difficult, with the only ways available being those of constant observation and the identification of distant metastases.

Enhanced CT, MRI, or Ga-68 DOTATATE PET/CT represent the best imaging methods for describing the tumor, being crucial in establishing a proper surgical strategy.

Prior to the surgery, the patient must undergo a complete cardiological, endocrinological, and, in some cases, neurological examination. Moreover, it is essential to establish whether we are dealing with a secretory paraganglioma by measuring the catecholamine levels in blood and urine.

Because paragangliomas are in the vast majority of cases highly vascularized tumors, measures must be considered for bleeding control such as embolization or ligation of the superior thyroid artery and, in some cases, even the temporary ligation of the external carotid artery.

The surgical approach, whether transoral endoscopic, whether external or mixed, is decided after considering all the factors, such as size, localization, bleeding control, patient health, equipment, and surgeon experience.

In cases when the paraganglioma proves to be malignant, radiotherapy can be considered.

## Data Availability

No new data were created or analyzed in this study. Data sharing is not applicable to this article.
